# AIDS-associated Kaposi's sarcoma is linked to advanced disease and high mortality in a primary care HIV programme in South Africa

**DOI:** 10.1186/1758-2652-13-23

**Published:** 2010-07-08

**Authors:** Kathryn M Chu, Gcina Mahlangeni, Sarah Swannet, Nathan P Ford, Andrew Boulle, Gilles Van Cutsem

**Affiliations:** 1 Médecins Sans Frontières, Braamfontein, Johannesburg, South Africa; 2 Médecins Sans Frontières, Khayelitsha, Cape Town, South Africa; 3 Infectious Disease Epidemiology Unit, School of Public Health and Family Medicine University of Cape Town, Observatory, Cape Town, South Africa

## Abstract

**Background:**

AIDS-associated Kaposi's sarcoma is an important, life-threatening opportunistic infection among people living with HIV/AIDS in resource-limited settings. In western countries, the introduction of combination antiretroviral therapy (cART) and new chemotherapeutic agents has resulted in decreased incidence and improved prognosis of AIDS-associated Kaposi's sarcoma. In African cohorts, however, mortality remains high. In this study, we describe disease characteristics and risk factors for mortality in a public sector HIV programme in South Africa.

**Methods:**

We analysed data from an observational cohort study of HIV-infected adults with AIDS-associated Kaposi's sarcoma, enrolled between May 2001 and January 2007 in three primary care clinics. Paper records from primary care and tertiary hospital oncology clinics were reviewed to determine the site of Kaposi's sarcoma lesions, immune reconstitution inflammatory syndrome stage, and treatment. Baseline characteristics, cART use and survival outcomes were extracted from an electronic database maintained for routine monitoring and evaluation. Cox regression was used to model associations with mortality.

**Results:**

Of 6292 patients, 215 (3.4%) had AIDS-associated Kaposi's sarcoma. Lesions were most commonly oral (65%) and on the lower extremities (56%). One quarter of patients did not receive cART. The mortality and lost-to-follow-up rates were, respectively, 25 (95% CI 19-32) and eight (95% CI 5-13) per 100 person years for patients who received cART, and 70 (95% CI 42-117) and 119 (80-176) per 100 person years for patients who did not receive cART. Advanced T stage (adjusted HR, AHR = 5.3, p < 0.001), advanced S stage (AHR = 5.1, p = 0.008), and absence of chemotherapy (AHR = 2.4, p = 0.012) were associated with mortality.

Patients with AIDS-associated Kaposi's sarcoma presented with advanced disease and high rates of mortality and loss to follow up. Risk factors for mortality included advanced Kaposi's sarcoma disease and lack of chemotherapy use. Contributing factors to the high mortality for patients with AIDS-associated Kaposi's sarcoma likely included late diagnosis of HIV disease, late accessibility to cART, and sub-optimal treatment of advanced Kaposi's sarcoma.

**Conclusions:**

These findings confirm the importance of early access to both cART and chemotherapy for patients with AIDS-associated Kaposi's sarcoma. Early diagnosis and improved treatment protocols in resource-poor settings are essential.

## Background

AIDS-associated Kaposi's sarcoma (AIDS-KS) is an important, life-threatening opportunistic infection among people living with HIV/AIDS in resource-limited settings. Treatment with combination antiretroviral treatment (cART) has led to a sharp decline in AIDS-KS incidence and mortality in European and North American cohorts [1-6]. Combination ART has also resulted in regression of Kaposi's sarcoma (KS) disease and even complete remission of KS lesions [7-9]. Adjuvant systemic chemotherapy appears to improve outcomes in advanced cases [10,11], particularly with newer chemotherapeutic agents, such as liposomal anthracyclines and taxanes, which have improved efficacy and tolerability compared with older drugs, such as bleomycin, doxorubicin, vincristine, vinblastine or adriamycin [12],

However, in sub-Saharan Africa, where cART is still not widely available and chemotherapy is very limited, KS mortality remains high [12]. In this study, we describe disease characteristics and risk factors for mortality in patients with AIDS-KS in a routine HIV programme in South Africa.

### Study site

We focused on three primary care HIV clinics in Khayelitsha, a poor township (population c.500,000) located in Cape Town, South Africa, with an adult antenatal prevalence of HIV of 33% [13]. Combination ART is provided by the provincial Department of Health with support from Médecins Sans Frontières (MSF). All patients were started on either nevirapine- or efavirenz-based triple therapy according to provincial treatment protocols.

At their initial visit, patients were examined for evidence of opportunistic infections, including AIDS-KS. Diagnoses were made clinically, and protocols recommended that those with AID-KS be started on cART irrespective of CD4 count, and those with advanced or extensive disease be referred to hospital oncology services for consideration of chemotherapy or radiotherapy.

## Methods

Our study included HIV-infected individuals enrolled between May 2001 and January 2007. Children (<18 years) were excluded. A chart review of patients with AIDS-KS was conducted to describe stage, anatomic distribution and treatment. Initial KS stage was determined using the AIDS Clinical Trials Group staging system that classifies tumour (T), immune (I) and systemic illness (S) status into good and poor risk [14]. KS immune reconstitution inflammatory syndrome (IRIS) was defined as worsening of KS disease soon after cART initation [15]. Disseminated cutaneous lesions were defined as 25 or more external lesions or the appearance of 10 or more new lesions over one month.

Chemotherapeutic agents used included bleomycin, vinblastine, vincristine, etoposide, cyclophosamide, adriamyacin and prednisone. There were no standard chemotherapeutic regimens; some patients received monotherapy, while others were treated with multiple drugs. An average of 6.5 cycles (range 1-20) was given to patients who received chemotherapy. Biopsies to confirm histology were performed only if the clinical diagnosis of KS was questionable (3%, seven patients).

Baseline characteristics, cART use and survival outcomes were extracted from an electronic database maintained for routine monitoring and evaluation. Survival outcomes were defined as death, loss to follow up (LTFU), transferred, or alive and on treatment, and were censored on 31 December 2007. Patient time to LTFU and death were calculated from date of AIDS-KS diagnosis. Patients were defined as LTFU if their last clinic visit occurred more than three months prior to 31 December 2007 and were censored at their last contact date. Transfers were censored at the transfer date. The national death registry and local hospital records were used to confirm vital status.

Cox proportional hazard models were built to model determinants of mortality and the Kaplan-Meier method was used to describe survival. A sensitivity analysis was performed combining patients lost to follow up with those confirmed dead to model determinants of mortality. Variables considered in the analysis included age, gender, baseline CD4 count (cells/mm^3^), KS stage, use of cART, chemotherapy and radiotherapy. All variables were included in the multivariate models, given their clinical plausibility. Statistical analysis was performed using STATA 11 (College Station, TX, USA).

Ethics approval for the study was obtained from the University of Cape Town and the University of Stellenbosch, South Africa.

## Results

Of 6292 adults enrolled in the HIV clinics in Khayelitsha during the study period, 215 (3.4%) had AIDS-KS. In total,189 (88%) charts were available for review. At the time of diagnosis, median age was 34 years (IQR 29-41 years), median CD4 count was 82 (IQR 31-174) cells/mm^3^, and 77 (41%) were female. The most common KS lesions were oral (65%) and on the lower extremities (56%). Of patients started on cART, seven (5%) had symptoms consistent with KS IRIS. At diagnosis, 124 patients (69%) were T1 stage, 149 (82%) were S1 stage, and eight (4%) were not staged (Table 1).

**Table 1:** Demographic and disease characteristics of AIDS-KS patients

Males	112 (59)
Age at time of AIDS-KS diagnosis, years	34(29-41)
Median baseline CD4+ count, cells/mm^3^	82 (31-174)
Follow-up time, months	278( 38-909)
Disseminated Cutaneous Lesions	72 (38)
IRIS	7 (5)
Site of Kaposi's sarcoma Lesions	
Oral	122 (65)
Head	78 (41)
Trunk	68 (36)
Upper Extremity	63 (33)
Lower Extremity	105 (56)
Lymphadenopahthy	42 (22)
Lymphoedema	42 (22)
Gastrointestinal Lesions**	4 (2)
Lung Lesions**	37 (20)
KS Stage	
Unstaged	8 (4)
T0	57 (31)
T1	124 (69)
S0	32 (18)
S1	149 (82)

Continous variables are given as medians (interquartile range). Ordinal and discrete variables are given as n(%).

Follow-up time from time of AIDS-KS diagnosis to survival outcome (death, loss-to-follow up, or censor)

IRIS, immune reconstitutioninflammatory syndrome. Denominator n = 137 for patients on cART only

** Suspected cases

More than a quarter (52, 27%) of patients did not receive cART. Fifty-five (29%) patients received chemotherapy, and 45 (24%) received radiotherapy. Median follow-up time was 278 days (IQR 45-747). Observation time totalled 234 person years. The mortality and lost-to-follow-up rates were, respectively, 29 (95% CI 23-37) and 16 (95% CI 11-22) per 100 person years for all patients, 25 (95% CI 19-32) and six (95% CI 3-10) per 100 person years for patients who received cART, and 70 (95% CI 42-117) and 119 (80-176) per 100 person years for patients who did not receive cART.

In multivariate analysis, advanced T stage (adjusted HR, AHR = 5.3, p < 0.001), advanced S stage (AHR = 5.1, p = 0.008), and lack of chemotherapy use (AHR = 2.4, p = 0.012) were associated with mortality (Table 2). In a sensitivity analysis that combined patients LTFU with those confirmed dead, lack of cART was strongly associated in mortality (AHR = 4.0, p < 0.001) (Table 3).

**Table 2:** Associations with mortality in AIDS-KS patients

Mortality						
	Unadjusted			Adjusted		
	**HR**	**95% CI**	**P**	**HR**	**95% CI**	**P**
	
Gender						
Male	1.0					
Female	1.0	(0.6-1.7)	0.863	1.3	(0.7-2.2)	0.399
Age						
≤35 years	1.0					
>35 years	1.2	(0.7-2.0)	0.430	1.6	(0.9-2.8)	0.107
Baseline CD4 count						
≤100 cells/μl	1.0					
>100 cells/μl	0.5	(0.3-0.9)	0.025	0.8	(0.5-1.5)	0.556
KS Stage						
T0	1.0					
T1	3.5	(1.8-6.8)	<0.001	5.3	(2.7-10.6)	<0.001
S0	1.0					
S1	5.7	(1.8-18-1)	0.003	5.1	(1.5-17.0)	0.008
cART						
Yes	1.0					
No	1.5	(0.8-2.8)	0.172	1.4	(0.7-2.8)	0.355
Chemotherapy						
Yes	1.0					
No	1.2	(0.7-2.2)	0.482	2.4	(1.2-4.8)	0.012
Radiation Therapy						
Yes	1.0					
No	1.3	(0.7-2.3)	0.398	1.1	(0.6-2.1)	0.668

KS, Kaposi's sarcoma. HR, Hazards ratio. cART, combination antiretroviral therapy

**Table 3:** Associations with mortality and lost to Follow-up in AIDS-KS patients

	Unadjusted			Adjusted		
	**HR**	**95% CI**	**P**	**HR**	**95% CI**	**P**

Gender						
Male	1.0					
Female	1.1	(0.7-1.6)	0.745	1.0	(0.6-1.6)	0.985
Age						
≤35 years	1.0					
>35 years	1.2	(0.8-1.7)	0.435	1.1	(0.7-1.8)	0.614
Baseline CD4 count						
≤100 cells/μl	1.0					
>100 cells/μl	0.6	(0.4-0.9)	0.012	0.7	(0.4-1.2)	0.194
KS Stage						
T0	1.0					
T1	2.4	(1.6-3.8)	<0.001	3.3	(2.0-5.5)	<0.001
S0	1.0					
S1	3.2	(1.5-6.6)	0.002	2.4	(1.1-5.2)	0.021
cART						
Yes	1.0					
No	3.4	(2.2-5.3)	<0.001	4	(2.4-6.6)	<0.001
Chemotherapy						
Yes	1.0					
No	1.3	(0.8-2.1)	0.333	1.9	(1.1-3.5)	0.025
Radiation Therapy						
Yes	1.0					
No	1.4	(0.9-2.3)	0.168	1.3	(0.8-2.2)	0.356

KS,Kaposi sarcoma. HR, Hazards Ratio. cART, combination antiretroviral therapy

**Figure 1:**
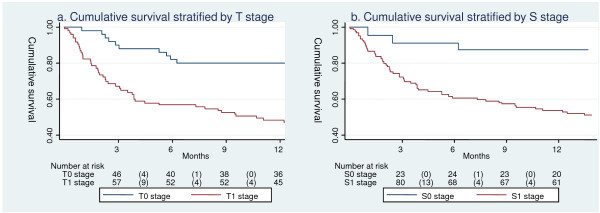
**Cumulative survival by T and S stages**.

**Figure 2:**
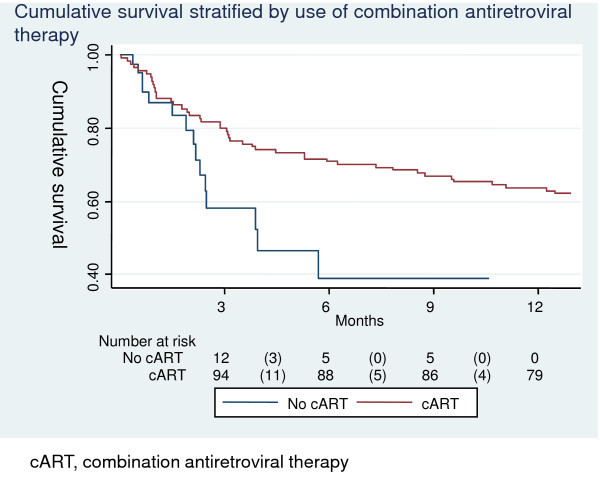
**Cumulative survival by use of combination antiretroviral therapy**.

Cumulative one-year survival of AIDS-KS patients stratified by T and S stages are shown in Figure 1. Figure 2 shows cumulative one-year survival stratified by cART. Overall cumulative survival at one year was 60% (95% CI 51-67%), 64% (95% CI 54-72%) for patients on cART, and 39% (95% CI 18-60%) for patients who did not receive cART. The cumulative incidence of LTFU at one year was 23% (95% CI 17-32), 7% (95% CI 2-14%) for patients on cART, and 79% (95% CI 60-94%) for patients not on cART.

Our study found that majority of patients presented with advanced stage KS; more than half of patients were diagnosed with T1S1 disease, a much higher proportion than reported from resource-rich countries [16]. Oral lesions and disseminated cutaneous lesions were common in our study and consistent with the severity of disease at presentation.

Half of AIDS-KS patients died or were lost to follow up in the first year. A substantial proportion of those lost to follow up are likely to have died: a recent meta-analysis has reported that 40% of patients lost to follow up are in fact dead [17]. Combination ART is protective against AIDS-KS mortality [12]. Unfortunately, one-quarter of patients in our cohort were not on cART. This may have been due to high pre-ART mortality due to late presentation, as well as high pre-ART loss to follow up. When LTFU was included with confirmed deaths, lack of cART became strongly associated with mortality because most of these patients likely died before they could start cART. Improved early diagnosis, access to cART, and retention in care for patients with AIDS-KS are needed.

Baseline CD4 count of less than 100 cells/mm^3 ^was associated with mortality on univariate analysis, but not on multivariate analysis. This is likely because the effects of advanced KS disease (T1 and S1 stages) were much stronger than CD4 count. Advanced T and S stage were strongly associated with mortality, while chemotherapy and cART use were associated with increased survival. While guidelines recommend treating advanced stage KS with chemotherapy, a proportion of patients did not receive it.

Liposomal drugs, the most effective treatment for advanced AIDS-KS are poorly available in sub-Saharan Africa due to their high cost. Improved studies on the effectiveness of accessible chemotherapy regimens and related side effects in resource-limited settings are needed. However, even in resource-rich countries, T1S1 disease is associated with increased mortality (53% survival at three years) despite better access to treatment [16]. Earlier diagnosis of HIV and AIDS-KS are imperative to improve survival.

Our study has several limitations. KS cases were identified through an electronic database of diagnoses recorded as part of routine monitoring in a large-scale cART programme. Additional cases may have been missed, or early cases may have resolved spontaneously on cART without being recorded. Charts for 12% of KS cases could not be located. Follow-up times were variable. The high rate of LTFU among patients not on cART likely led to an underestimation of the beneficial effects of cART, as indicated by our post hoc sensitivity analysis. Finally, other risk factors for mortality, such as other opportunistic infections like tuberculosis, were not considered.

## Conclusions

In conclusion, our study details the late presentation of patients with AIDS-KS, the high mortality and loss to follow up at one year, the relationship of advanced KS disease to mortality, and the incomplete access to chemotherapy for those with advanced disease. Contributing factors likely include late diagnosis of HIV disease, late accessibility to cART, and sub-optimal treatment of advanced KS.

These findings confirm the importance of early access to both cART and chemotherapy for patients with AIDS-associated KS. KS is the most common HIV-related malignancy and an important contributor to AIDS-related mortality. Early diagnosis and improved treatment protocols in resource-poor settings are essential.

## Competing interests

The authors declare that they have no competing interests.

## Authors' contributions

KC was responsible for the overall design, analysis and writing of the paper. GM and GVC wrote the first draft of the study protocol. GM, SS, AB and GVC contributed to the data collection or analysis. NF, AB and GVC contributed to the concept, intellectual content and writing of the paper. The final version of the manuscript was seen and approved by all authors. The corresponding author held the final responsibility for submitting the manuscript for publication.
